# HIF-1α is required for hematopoietic stem cell mobilization and 4-prolyl hydroxylase inhibitors enhance mobilization by stabilizing HIF-1α

**DOI:** 10.1038/leu.2015.8

**Published:** 2015-02-03

**Authors:** C E Forristal, B Nowlan, R N Jacobsen, V Barbier, G Walkinshaw, C R Walkley, I G Winkler, J P Levesque

**Affiliations:** 1 Blood and Bone Diseases Program, Mater Research Institute, University of Queensland, Woolloongabba, Queensland, Australia; 2 School of Medicine, University of Queensland, Brisbane, Queensland, Australia; 3 Fibrogen Inc., San Francisco, CA, USA; 4 Stem Cell Regulation Unit, St Vincent's Institute and University of Melbourne, St Vincent's Hospital, Fitzroy, Victoria, Australia

## Abstract

Many patients with hematological neoplasms fail to mobilize sufficient numbers of hematopoietic stem cells (HSCs) in response to granulocyte colony-stimulating factor (G-CSF) precluding subsequent autologous HSC transplantation. Plerixafor, a specific antagonist of the chemokine receptor CXCR4, can rescue some but not all patients who failed to mobilize with G-CSF alone. These refractory poor mobilizers cannot currently benefit from autologous transplantation. To discover alternative targetable pathways to enhance HSC mobilization, we studied the role of hypoxia-inducible factor-1α (HIF-1α) and the effect of HIF-1α pharmacological stabilization on HSC mobilization in mice. We demonstrate in mice with HSC-specific conditional deletion of the *Hif1a* gene that the oxygen-labile transcription factor HIF-1α is essential for HSC mobilization in response to G-CSF and Plerixafor. Conversely, pharmacological stabilization of HIF-1α with the 4-prolyl hydroxylase inhibitor FG-4497 synergizes with G-CSF and Plerixafor increasing mobilization of reconstituting HSCs 20-fold compared with G-CSF plus Plerixafor, currently the most potent mobilizing combination used in the clinic.

## Introduction

The cytokine granulocyte colony-stimulating factor (G-CSF) is the main agent to mobilize hematopoietic stem cells (HSCs) for transplantation. Administered daily at doses of 10 μg/kg, G-CSF mobilizes hematopoietic stem and progenitor cells (HSPCs) from the bone marrow (BM) into the circulation. In most healthy allogeneic donors, CD34^+^ HSPCs are robustly mobilized after 5 days of G-CSF treatment and blood aphaeresis from day 5 is sufficient to reach the minimum threshold of 2 × 10^6^ CD34^+^ cells/kg body weight to ensure rapid hematopoietic reconstitution. However in the autologous setting, 20–60% of chemotherapy-treated patients fail to reach this minimal threshold in response to G-CSF, precluding transplantation^1^ particularly in patients with prior radiotherapy and chemotherapy.^1, 2^ The chemotactic interaction between the chemokine CXCL12 and its receptor CXCR4 is pivotal to HSPC retention within the BM^3, 4^ and is weakened by G-CSF treatment causing mobilization.^5, 6, 7^ Additional inhibition of the CXCL12:CXCR4 interaction with specific small synthetic inhibitors such as Plerixafor (previously called AMD3100) synergistically enhances HSPC mobilization in response to G-CSF in humans and mice.^8^ The synergistic effect of Plerixafor has been confirmed in large clinical trials with multiple myeloma and non-Hodgkin lymphoma patients eligible for autologous HSC transplantation who previously failed to mobilize in response to G-CSF alone. Plerixafor injected daily 1 h before blood aphaeresis from day 4 of G-CSF administration enables approximately 70–80% patients who previously failed to mobilize in response to G-CSF alone to reach 2 × 10^6^ CD34^+^ cells/kg threshold.^9, 10^ However, the remaining 20–30% of patients who failed to mobilize with G-CSF alone still fail to mobilize with G-CSF and Plerixafor.^9, 10^ It is therefore important to further understand the mechanisms of HSC mobilization to identify novel therapeutics to overcome mobilization failure in a larger proportion of patients.

The hypoxia sensing pathway is activated in the BM of mice mobilized with G-CSF.^11^ Extensive myeloid progenitor proliferation in response to G-CSF is thought to enhance O_2_ consumption increasing overall BM hypoxia, which in turn stabilizes the O_2_-labile protein hypoxia-inducible factor-1α (HIF-1α) in mobilized BM.^11^ HIF-1α is a transcription factor that regulates metabolic adaptation and cell responses to hypoxia.^12, 13, 14^ Considering the critical role of HIF-1α in regulating HSC proliferation, self-renewal and homing to the BM,^15, 16, 17, 18^ we investigated its role in HSPC mobilization in mice.

## Materials and methods

### Mice

C57BL/6 mice were purchased from the Australian Resource Centre, Perth, Australia; B6.129-*Hif1a*^tm3Rsjo^/J (*Hif1a*^flox/flox^) mice from the Jackson Laboratory (Bar Harbor, ME, USA). *Scl*-CreER transgenic mice expressing a tamoxifen-inducible Cre recombinase driven by a HSC-specific *Scl* gene enhancer,^19^ B6-Gt(ROSA)26^Sortm1(EYFP)Cos/J^ (abbreviated as R26R^YFP^) with a loxP-flanked STOP sequence preceding an enhanced yellow fluorescent protein (YFP) reporter gene inserted into the genetrap ROSA26 locus^20^ were backcrossed at least 10 times in C57BL/6 background. Mouse genotyping is described in Supplementary Methods.

### Induction of the *Scl*-CreER transgene with tamoxifen

*Scl*-CreER mice were gavaged daily with tamoxifen-free base diluted in peanut oil containing 10% ethanol for 3 days to induce the *Scl*-CreER transgene. For mobilization experiments with G-CSF, G-CSF was administered for 3 days beginning on the final day of tamoxifen gavaging unless otherwise stated. For mobilization experiments with Plerixafor, Plerixafor was administered intraperitoneally 1 week following the final day of tamoxifen induction, 1 h before harvest. Following treatments, mice were then euthanized by cervical dislocation and BM and bones harvested. For migration assay and CXCR4 staining, mice were harvested 1 week following the final day of tamoxifen induction.

### Mobilization

All experiments were performed on 9- to 12-week-old male mice. Recombinant human G-CSF was injected twice daily subcutaneously at 125 μg/kg and Plerixafor (AMD3100 octohydrochloride) intraperitoneally at 10 mg/kg as described.^21^ Tissues were harvested 1 h after Plerixafor administration. FG-4497 was injected daily intraperitoneally at 20 mg/kg. Control mice were injected with an equivalent volume of saline. Tissues were harvested on the morning following the last G-CSF injection, or 1 h following Plerixafor injection.

For studies with additional 4-prolyl hydroxylase domain enzyme (PHD) inhibitors, G-CSF was administered twice daily subcutaneously for 4 consecutive days. PHI-1 and PHI-2 were administered daily by oral gavage at 20 mg/kg for the last 3 days of the mobilization regimen.

### Tissue harvesting

Mice were anesthetized with isofluorane and approximately 1 ml of blood collected into heparinized tubes by cardiac puncture before cervical dislocation. Femoral BM was flushed and spleens dissociated between the frosted ends of two glass slides in phosphate-buffered saline containing 2% fetal calf serum (FCS) for further analyses. For flow-cytometry analyses, red cells were lysed from blood samples as previously described.^22^ Spleens were harvested, weighed, dissociated in 10 ml phosphate-buffered saline with 2% FCS. RNA from the endosteum was isolated from femurs as previously described.^23^

### Western blotting

Following treatment of mice with Saline, G-CSF or FG-4497, BM cells from one femur were flushed with 1 ml of urea cell lysis buffer containing complete protease inhibitors (Roche, Basel, Switzerland) supplemented with 200 μM phenylmethylsulfonyl fluoride.^16^ Quantitative western blots with rabbit anti-mouse HIF-1α antibody were performed using the Odyssey Infra-Red Imaging System (Li-COR Bioscience, Lincoln, NE, USA) as described previously.^11, 16^

### Colony assays

In all, 10 μl whole blood or splenocytes from 1/1000 of a whole spleen were seeded in 35-mm petri dishes and covered with 1 ml IMDM supplemented with 1.6% methylcellulose, 35% FCS and optimal concentrations of mouse IL-3, IL-6 and soluble kit ligand as described previously.^24^ When results are reported as proportion of mobilized cells versus whole BM content, we considered that one femoral BM represents 5.6% of total mouse BM and that the total volume of blood is 0.08 ml per gram of body weight.^25, 26^

### Flow cytometry

Antibody staining for expression of α4 and α5 integrins, PSGL1, CXCR4, binding of P- and E-selectins and for HSPC cell cycling are described in Supplementary Methods.

### Quantitative real-time reversed transcribed PCR

Quantitative real-time reversed transcribed PCR (qRT-PCR) to confirm *Hif1a* gene deletion in HSPC and measure *Cxcr4*, *Cxcr7* and *Cxcl12* mRNA is described in Supplementary Methods.

### Competitive repopulation assays

The content of mobilized blood samples in competitive repopulating HSC was determined in competitive repopulation assays as previously described.^22, 24^ Briefly, lethally irradiated recipient congenic B6.SJL CD45.1^+^ female mice were transplanted with 200 000 competitive whole BM cells from untreated B6.SJL CD45.1^+^ mixed with 20 μl blood aliquots from 6 pooled mobilized CD45.2^+^ C57BL/6 donor mice. CD45.2/CD45.1 chimerism was measured in blood at 8, 12 and 16 weeks post transplant in myeloid, B and T lineages by flow cytometry. Content in repopulating units was calculated as previously described.^21, 27^

### Migration assays

Five million whole BM cells from SclCreER R26R^YFP/YFP^ *Hif1a*^WT/WT^ or *Scl*CreER R26R^YFP/YFP^ *Hif1a*^fl/fl^ mice were plated in each 5 μM pore transwell inserts (Corning, Cambridge, MA, USA) in IMDM supplemented with 10% FCS in duplicate. CXCL12 was added to wells at a concentration of 250 ng/ml. After 4 h at 37 °C, the percentage of YFP^+^ and YFP^−^ Lin^−^Sca1^+^Kit^+^ cells that had transmigrated to the lower chamber was determined by flow cytometry after addition of 1000 fluorescent counting beads (Trucount tubes BD, Franklin Lakes, NJ, USA) in both insert and input cells. For chemotaxis assays with BM cells from wild-type mice treated with FG-4497, assays were set up as described above except that 40 μM FG-4497 was added in both top and bottom chambers containing BM cells from FG-4497-treated mice, whereas an equivalent volume of DMSO (0.2% final) was added in both chambers containing BM cells from saline-treated mice.

### Statistical analyses

Differences between treatment groups were calculated using a two-tailed *t*-test, paired *t*-test, ANOVA with Bonferroni correction or non-parametric Mann–Whitney test or Dunn's multiple comparison test depending on distribution normality. A value of *P*<0.05 was considered as significant. Data are presented as mean±standard deviation.

## Results

### *Hif1a* gene deletion in HSPCs impairs their mobilization in response to G-CSF

**Figure 1:**
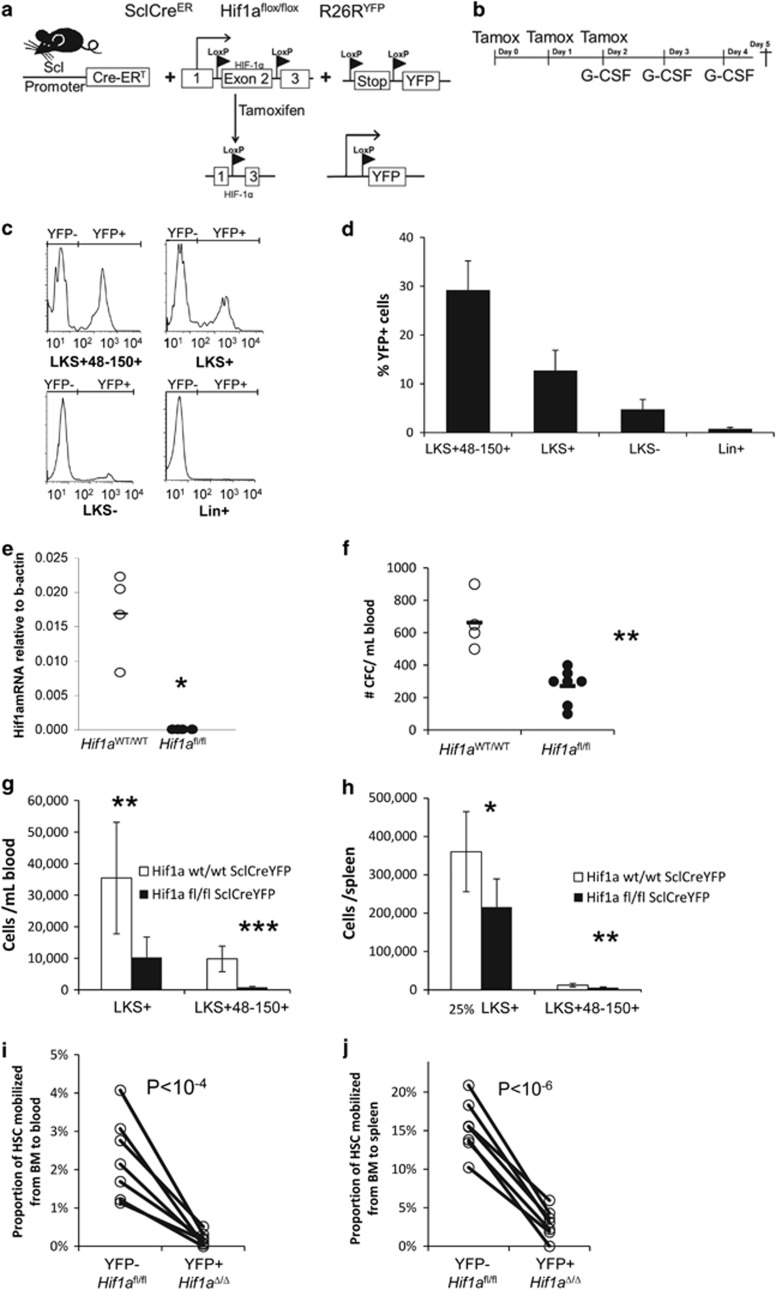
Deletion of *Hif1a* gene in HSPCs compromises HSPC mobilization in response to G-CSF. (**a**) Schematic representation of the tamoxifen-inducible Cre-dependant *Hif1a* deletion and YFP induction. Mice were mated to obtain two floxed *Hif1a* alleles (*Hif1a*^fl/fl^), with a Cre-inducible YFP reporter knocked in the Rosa26 gene trap locus (R26R^YFP^) and a tamoxifen-inducible Cre recombinase (CreER fusion protein) driven by the HSC-specific 6E5 enhancer fragment and promoter of the Scl gene (SclCreER). Tamoxifen specifically activates CreER in HSCs and consequently deletes the two *Hif1a* alleles and activates YFP expression. (**b**) Time course of CreER activation in HSPCs and mobilization with G-CSF. Tamoxifen was administered daily for 3 days and then mice were mobilized for 3 days with G-CSF twice daily. (**c, d**) Proportion of Lin^−^Sca1^+^Kit^+^ CD48^−^ CD150^+^ HSCs, Lin^−^Sca1^+^Kit^+^ HSPCs, Lin^−^Sca1^−^Kit^+^ myeloid progenitors and Lin^+^ leukocytes in which YFP is induced by CreER following a 3-day tamoxifen treatment in *Scl*CreER R26R^YFP/YFP^ *Hif1a*^fl/fl^ mice. (**e**) qRT-PCR showing levels of *Hif1a* mRNA in Lin^−^ Sca1^+^ Kit^+^ YFP^+^ HSPCs sorted from the BM of *Scl*CreER R26R^YFP/YFP^ *Hif1a*^fl/fl^ (*Hif1a*^fl/fl^) mice or *Scl*CreER R26R^YFP/YFP^ *Hif1a*^WT/WT^ (*Hif1a*^WT/WT^) mice and grown for 9 days in a colony assay. Results are relative to β-actin mRNA, each dot represents a separate mouse. (**f**) Number of CFCs mobilized into blood in *Scl*CreER R26R^YFP/YFP^ *Hif1a*^fl/fl^ mice and control *Scl*CreER R26R^YFP/YFP^ *Hif1a*^WT/WT^ mice. Each dot represents a separate mouse. (**g, h**) Number of phenotypic Lin^−^Sca1^+^Kit^+^CD48^−^CD150^+^ HSCs, Lin^−^Sca1^+^Kit^+^ HSPCs mobilized into blood (**g**) and spleen (**h**) in *Scl*CreER R26R^YFP/YFP^ *Hif1a*^fl/fl^ mice and control *Scl*CreER R26R^YFP/YFP^ *Hif1a*^WT/WT^ mice. Data are mean±s.d. of seven mice per group. *P-*values were calculated with a *t*-test. (**i**, **j**) Proportion of YFP^−^ (*Hif1a*^fl/fl^ non-deleted) and YFP^+^ (*Hif1a*^Δ/Δ^ deleted) HSCs in the blood (**i**) or spleen (**j**) over YFP^−^ or YFP^+^ HSCs remaining in the BM following a CreER induction with tamoxifen and a 3-day G-CSF treatment. Pairs of dots show results for YFP^−^ and YFP^+^ HSC within the same *Scl*CreER R26R^YFP/YFP^ *Hif1a*^fl/fl^ mouse. *P*-values were calculated with a paired *t*-test. Symbols for significance levels are *P<0.05; **0.001<*P*<0.01; ***P<0.001.

To test the hypothesis that HIF-1α has an important role in HSC mobilization, we established a mutant mouse strain in which both *Hif1a* gene alleles are specifically deleted and YFP reporter induced in a Cre-dependent manner^20^ in HSCs following treatment with tamoxifen (Figure 1a).^19^ Measurements of the Cre-induced YFP reporter by flow cytometry showed that a 3-day tamoxifen treatment of *Scl*CreER R26R^YFP/YFP^ *Hif1a*^fl/fl^ mice caused Cre activation in 30±9% of Lin^−^Sca1^+^Kit^+^CD48^−^CD150^+^ HSCs, 10±5% of Lin^−^Sca1^+^Kit^+^ HSPCs but was virtually undetected in Lin^−^Sca1^−^Kit^+^ myeloid/lymphoid progenitors or lineage-positive cells (Figures 1b and d). Therefore, a short tamoxifen treatment activates CreER specifically in the HSC compartment in these mice. To confirm *Hif1a* gene deletion, a primer pair was designed with the forward primer in the floxed exon 2 and the reverse primer in exon 3 of the *Hif1a* gene to quantify by qRT-PCR intact *Hif1a* mRNA. Lin^−^Sca1^−^Kit^+^ YFP^+^ HSPCs and Lin^−^Sca1^+^Kit^+^CD48^−^CD150^+^ HSCs were sorted 4 days after the end of the 3-day induction with tamoxifen and assessed for *Hif1a* mRNA content by qRT-PCR. Intact *Hif1a* mRNA was significantly reduced in HSCs and HSPCs from *Scl*CreER R26R^YFP/YFP^ *Hif1a*^fl/fl^ mice compared with *Scl*CreER R26R^YFP/YFP^ *Hif1a*^WT/WT^ mice (Supplementary Figure S1). We also plated sorted Lin^−^Sca1^−^Kit^+^ YFP^+^ HSPCs in colony assays in methylcellulose. After 9 days of culture, individual colonies were picked and RNA extracted. Intact *Hif1a* mRNA was undetectable in colonies from *Scl*CreER R26R^YFP/YFP^ *Hif1a*^fl/fl^ mice (Figure 1e). Together, these results demonstrate efficient deletion of the *Hif1a* gene in HSCs following tamoxifen gavage in *Scl*CreER R26R^YFP/YFP^ *Hif1a*^fl/fl^ mice.

*Scl*CreER R26R^YFP/YFP^ *Hif1a*^fl/fl^ mice and control *Scl*CreER R26R^YFP/YFP^ *Hif1a*^WT/WT^ mice were induced with tamoxifen for 3 days and then G-CSF for 3 days from the last day of tamoxifen induction (Figure 1b). Deletion of *Hif1a* gene in HSPCs significantly reduced G-CSF-induced mobilization of colony-forming cells (CFCs) to the blood (Figure 1f), and phenotypic HSPCs and HSCs to the blood and spleen of *Scl*CreER R26R^YFP/YFP^ *Hif1a*^fl/fl^ mice compared with control *Scl*CreER R26R^YFP/YFP^ *Hif1a*^WT/WT^ mice (Figures 1g and h). Decreased HSC mobilization following *Hif1a* deletion was not due to decreased number of HSCs in the BM as *Scl*CreER R26R^YFP/YFP^ *Hif1a*^fl/fl^ and control *Scl*CreER R26R^YFP/YFP^ *Hif1a*^WT/WT^ mice had similar numbers of HSCs per femur following deletion with tamoxifen and G-CSF treatment and similar total number of HSCs following summation of HSC numbers in total skeletal BM, blood and spleen (Supplementary Figure S2). As *Hif1a* was deleted from approximately 30% of HSCs in *Scl*CreER R26R^YFP/YFP^ *Hif1a*^fl/fl^ mice treated with tamoxifen and G-CSF, we took advantage of this mosaic deletion to calculate within each individual mouse the proportion of YFP^−^ (*Hif1a*^*fl*^ gene intact, not deleted) and YFP^+^ (*Hif1a* deleted) Lin^−^Sca1^+^Kit^+^ HSPCs mobilized to blood or spleen compared with those remaining within the BM. Within each individual mouse, the proportion of YFP^+^ *Hif1a*-deleted HSPCs that left the BM to mobilize into blood or spleen was 12-fold and 5-fold lower, respectively, than the proportion of YFP^-^ *Hif1a*-intact HSPCs mobilized to blood and spleen (Figures 1i and j). Therefore, *Hif1a* gene deletion in HSPCs impairs their mobilization in response to G-CSF.

### *Hif1a* deletion impairs mobilization induced by CXCR4 blockade

**Figure 2:**
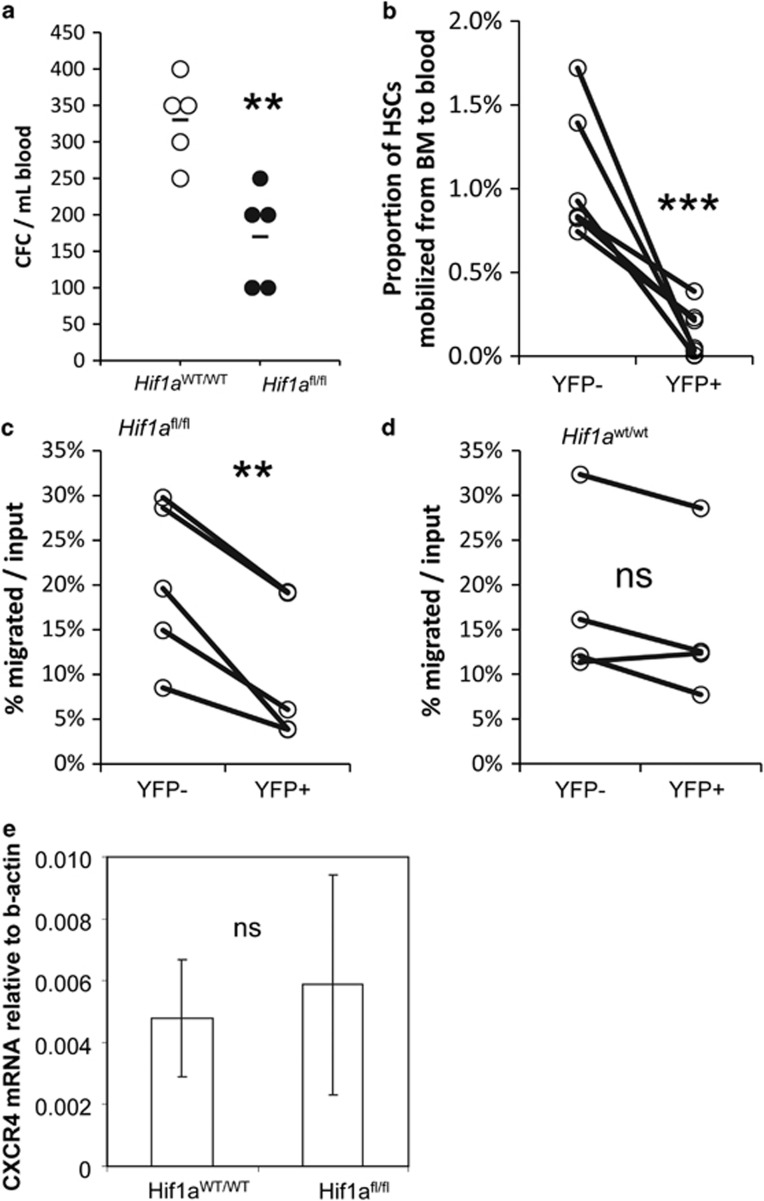
Deletion of *Hif1a* gene in HSPCs disrupts the CXCR4 signaling pathway. (**a**) Number of CFC per mL blood following a CreER induction with tamoxifen and a 1 h treatment with Plerixafor. Each dot represents a mouse. (**b**) Proportion of YFP^−^ (*Hif1a*^fl/fl^ non-deleted) and YFP^+^ (*Hif1a*^Δ/Δ^ deleted) Lin^−^Sca1^+^Kit^+^CD48^-^CD150^+^ HSCs in the blood over YFP^−^ or YFP^+^ HSCs remaining in the BM following a CreER induction with tamoxifen and a 1-h treatment with Plerixafor. Each pair of dots shows results from YFP^−^ and YFP^+^ HSCs within the same *Scl*CreER R26R^YFP/YFP^ *Hif1a*^fl/fl^ mouse. Significance was calculated with a paired *t*-test. (**c, d**) Proportion of YFP^−^ and YFP^+^ LSK HSPCs migrated toward CXCL12 as percentage of input YFP^−^ or YFP^+^ HSPCs. Pairs of dots show results for YFP^−^ and YFP^+^ HSCs within each single *Scl*CreER R26R^YFP/YFP^ *Hif1a*^fl/fl^ mouse (**c**) or control *Scl*CreER R26R^YFP/YFP^ *Hif1a*^wt/wt^ mouse (**d**). Significance were calculated with a paired *t*-test. (**e**) *Cxcr4* mRNA expression levels in Lin^−^Sca1^+^Kit^+^ YFP+ HSPCs sorted from the BM of *Scl*CreER R26R^YFP/YFP^ *Hif1a*^fl/fl^ (*Hif1a*^fl/fl^) mice or *Scl*CreER R26R^YFP/YFP^ *Hif1a*^WT/WT^ (*Hif1a*^WT/WT^) mice. Data are relative to *β-actin* mRNA. Data are mean±s.d. of 4–5 mice per group. Symbols for significance levels are **0.001<*P*<0.01; ***P<0.001.

We next evaluated the effect of *Hif1a* gene deletion on mobilization in response to the CXCR4 antagonist Plerixafor. *Scl*CreER R26R^YFP/YFP^ *Hif1a*^fl/fl^ mice and control *Scl*CreER R26R^YFP/YFP^ *Hif1a*^WT/WT^ mice were induced with tamoxifen for 3 days and then with a single dose of Plerixafor 1 week following the last day of tamoxifen induction. Deletion of *Hif1a* gene significantly reduced Plerixafor-induced mobilization of CFCs to the blood (Figure 2a). We calculated within each individual mouse the proportion of YFP^−^ (*Hif1a* gene intact) and YFP^+^ (*Hif1a* deleted) Lin^−^Sca1^+^Kit^+^ HSPCs mobilized to blood versus the number of *Hif1a*-intact or *Hif1a*-deleted HSPCs remaining within the BM. Within each individual mouse, the proportion of YFP^+^ *Hif1a*-deleted HSPCs that mobilized into blood or spleen significantly reduced compared with the proportion of YFP^+^ *Hif1a*-intact HSPCs that mobilized to the blood (Figure 2b). This demonstrates that *Hif1a* gene deletion in HSPCs severely impairs mobilization in response to CXCR4 blockade.

To investigate whether *Hif1a* gene deletion disrupts the CXCR4/CXCL12 chemotactic signaling pathway, we performed a transmigration assay toward CXCL12. Whole BM cells were isolated from *Scl*CreER R26R^YFP/YFP^ *Hif1a*^fl/fl^ mice and *Scl*CreER R26R^YFP/YFP^ *Hif1a*^WT/WT^ mice induced with tamoxifen for 3 days, and deposited into transwells with 250 ng/ml CXCL12 in the bottom chamber. We calculated within each individual *Scl*CreER R26R^YFP/YFP^ *Hif1a*^fl/fl^ mouse, the proportion of YFP^−^ (*Hif1a* gene not deleted) and YFP^+^ (*Hif1a* deleted) Lin^−^Sca1^+^Kit^+^ HSPCs that migrated toward CXCL12. YFP^+^ (*Hif1a* deleted) HSPCs exhibited significantly reduced migration toward CXCL12 compared with YFP^-^ (*Hif1a* gene intact) HSPCs (Figure 2c). In control *Scl*CreER R26R^YFP/YFP^ *Hif1a*^WT/WT^ mice, migration of YFP^+^ and YFP^−^ HSPCs in response to CXCL12 was not significantly different (Figure 2d), suggesting that impaired migration toward CXCL12 was specifically due to *Hif1a* deletion.

As cell surface expression of CXCR4 is very low, we quantified *Cxcr4* mRNA by qRT-PCR on Lin^−^Sca1^−^Kit^+^ YFP^+^ HSPCs from *Scl*CreER R26R^YFP/YFP^ *Hif1a*^fl/fl^ mice and *Scl*CreER R26R^YFP/YFP^ *Hif1a*^WT/WT^ mice 4 days after a 3-day induction with tamoxifen. *Cxcr4* expression was however not altered by *Hif1a* deletion. We also tested for the expression of CXCR7, a related chemokine receptor previously shown to bind CXCL12 and dimerize and co-signal with CXCR4 on human CD34^+^ HSPCs.^28, 29^ Using an anti-CXCR7 monoclonal antibody cross-reactive for human and mouse CXCR7, as well as TaqMan primer probe set for mouse *Cxcr7* mRNA, we could not detect CXCR7 protein or mRNA in mouse BM HSPCs (data not shown), in agreement with expression array reported on www.biogps.org showing lack of expression in adult mouse HSCs.

### FG-4497 synergizes with G-CSF to enhance mobilization

**Figure 3:**
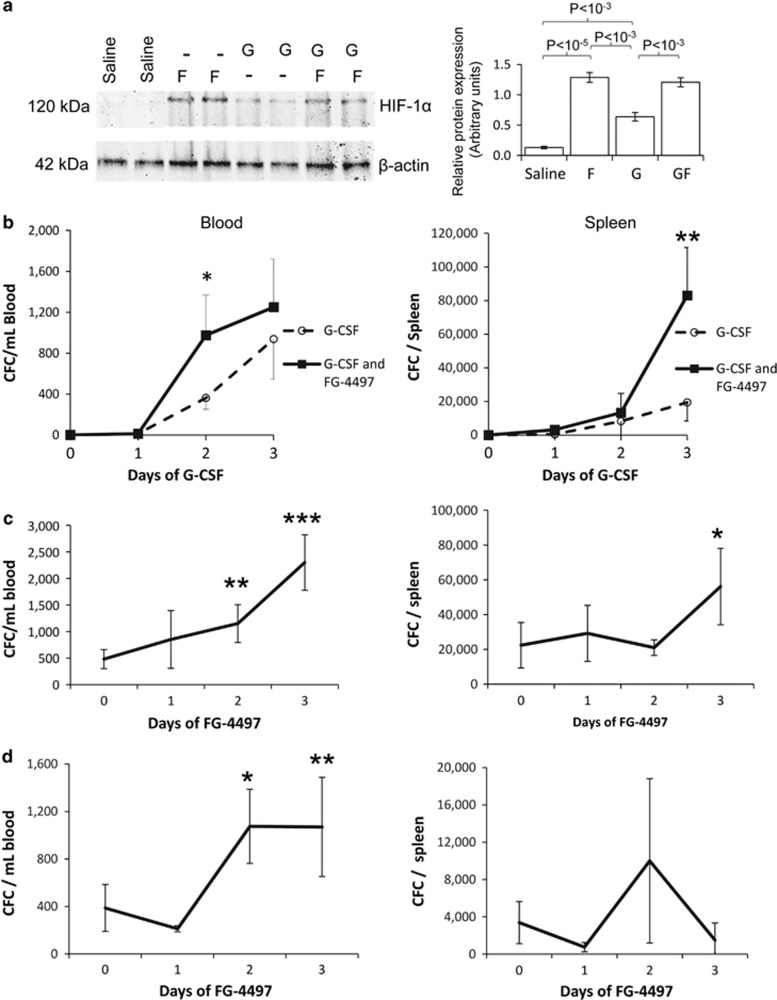
Effect of FG-4497 on HIF-1α protein and CFC mobilization. (**a**) BM cell lysates from mice treated with saline, FG-4497 for 3 days (F), G-CSF for 2 days (G) or both (GF) were western blotted for HIF-1α and β-actin. Each lane represents a different mouse. Histograms represent means±s.d. of three mice per treatment group. (**b**) Number of CFCs per ml blood and per spleen when mice were injected twice daily with G-CSF for indicated numbers of days and injected daily with FG-4497 for the last 3 days prior harvest. Significance levels are for each time point between groups treated with G-CSF alone or with G-CSF plus FG-4497. (**c**) Number of CFCs per ml blood and per spleen when mice were injected daily with FG-4497 for the indicated numbers of days and injected twice daily with G-CSF for 2 days prior harvest. Significance levels are relative to the group treated with G-CSF without FG-4497. (**d**) Number of CFCs per ml blood and per spleen when mice were injected daily with FG-4497 for indicated numbers of days and injected with Plerixafor for 1 h prior harvest. Significance levels are relative to the group treated with Plerixafor without FG-4497. Data are mean±s.d. of six mice per condition. Symbols for significance levels are *P<0.05; **0.001<*P*<0.01; ***P<0.001.

As HIF-1α is critical to HSPC mobilization in response to G-CSF or Plerixafor (Figures 1 and 2), we tested whether further stabilization of the HIF-1α protein, which is O_2_-labile, may enhance mobilization in response to G-CSF and Plerixafor. We injected wild-type mice with the PHD inhibitor FG-4497 to block HIF-1α proline hydroxylation in oxidative conditions and downstream recruitment of the ubiquitin ligase VHL and O_2_-dependant proteasomal degradation of HIF-1α.^16, 30, 31^ HIF-1α protein was very low in BM cell lysates from saline-injected animals and significantly increased following G-CSF treatment (Figure 3a) consistent with our previous findings.^11^ However, treatment with FG-4497 or the combination of FG-4497 with G-CSF further increased HIF-1α protein levels compared with G-CSF alone (Figure 3a).

We next treated C57BL/6 mice with G-CSF twice daily for 1–3 days together with the PHD inhibitor FG-4497 daily for the 3 days before tissue harvest. FG-4497 treatment on top of the 3 day G-CSF treatment increased CFCs mobilized per ml blood 2.5-fold following 2 days of G-CSF, and 6-fold the number of CFCs mobilized per spleen compared with G-CSF treatment alone (Figure 3b). Of note, 3-day treatment with FG-4497 alone without G-CSF did not induce CFC mobilization into the blood compared with control mice injected with vehicle (not shown).

To evaluate the effect of FG-4497 treatment duration, FG-4497 was administered for 1–3 days and G-CSF for the last 2 days before tissue sampling (Figure 3c). CFC mobilization into blood and spleen in response to 2 days of G-CSF progressively increased with the duration of FG-4497 treatment.

Of note FG-4497 did not enhance mobilization of Lin^−^Sca1^+^Kit^+^ HSPCs to blood and spleen in SclCreER R26R^YFP/YFP^ *Hif1a*^fl/fl^ mice treated with tamoxifen whereas it further enhanced mobilization of HSPCs in control SclCreER R26R^YFP/YFP^ *Hif1a*^WT/WT^ mice (Supplementary Figure S3). Therefore, the enhancing effect of FG-4497 on HSPC mobilization is specifically mediated by HIF-1α protein.

### FG-4497 synergizes with Plerixafor to enhance CFC mobilization

Since mobilization with Plerixafor peaks 1 h post injection,^8^ this time point was used together with daily administration of FG-4497 for 1–3 days before killing (Figure 3d). CFCs mobilization into blood in response to Plerixafor was significantly increased by a 2–3 day treatment with FG-4497 treatment demonstrating synergy between CXCR4 inhibition and PHD inhibition. As mobilization in response to Plerixafor is very rapid, there was no significant mobilization of HSPCs to the spleen 1 h following Plerixafor treatment with or without FG-4497.

### FG4497 does not increase mobilization by altering HSPC cycling or survival

As we have previously reported that a 7-day FG-4497 treatment inhibits HSPC proliferation *in vivo* in mice in steady state,^16^ we first evaluated 5-bromodeoxyuridine (BrdU) incorporation over 3 days and Ki67 antigen expression in BM HSPC from mice treated with saline, FG-4497 for 3 days, G-CSF for 2 days or G-CSF for 2 days together with FG-4497 for 3 days (Supplementary Figure S4A). A 3-day treatment with FG-4497 was not sufficiently long to significantly increase the proportion of Lin^−^Sca1^+^Kit^+^ HSPCs or Lin^−^Sca1^+^Kit^+^CD48^-^CD150^+^ HSCs in phase G_0_, or to counteract the increased cycling caused by 2 days of G-CSF (*P*<0.05 for HSCs and HSPCs in G_0_ in Saline vs G-CSF groups; or FG-4497 vs G-CSF plus FG4497 groups; but *P*>0.4 for G-CSF vs G-CSF plus FG4497 groups). Therefore, this short FG-4497 treatment did not increase HSPC mobilization by altering their cycling. We also measured the effect of FG-4497 on HSPC apoptosis. In overnight cultures of BM cells with 10 ng/ml Kit ligand, addition of 40 μM FG-4997, a concentration that stabilizes HIF-1α in cultured HSPC,^32^ did not alter the proportion of live non-apoptotic annexin V-negative, 7-aminoactinomycin D-negative HSPCs (Supplementary Figure S4B). Finally, FG-4497 treatment alone did not alter number of Lin^−^Kit^+^Sca1^+^ HSPC in the femoral BM compared with saline-treated mice. In contrast, the combination of 2 days G-CSF with 3 days FG-4497 reduced HSPC number in the BM significantly more than G-CSF alone treatment (Supplementary Figure S4C). Therefore, increased mobilization with G-CSF plus FG-4497 is not due to an increase in HSPC number in the BM. Rather, the decrease in the number of HSPCs in the BM may reflect better mobilization efficiency.

### FG-4497 alters CXCR4 distribution and signaling in G-CSF-mobilized mice

**Figure 4:**
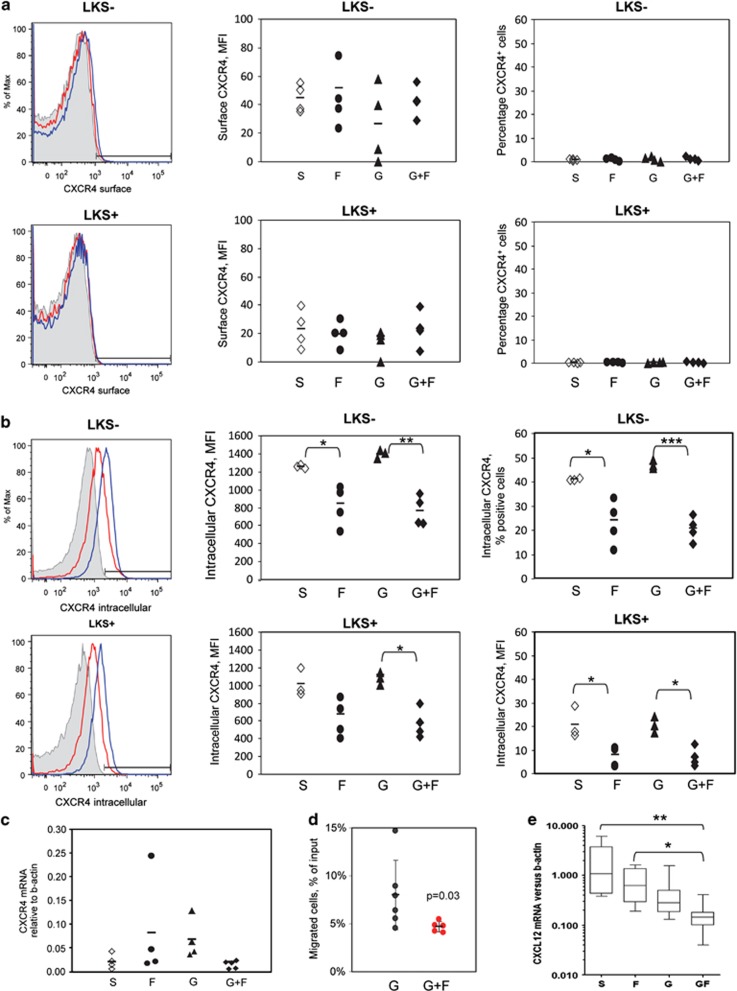
Effect of FG-4497 and G-CSF treatment on CXCR4 expression and cell migration. (**a**) Cell surface expression of CXCR4 on Lin^−^Sca1^−^Kit^+^ BM HPCs (LKS+) and Lin^−^Sca1^+^Kit^+^ HSPCs (LKS+) from mice treated for 2 days with saline (S), or with FG-3347 for 3 days (F), or with G-CSF for 2 days (G), or the combination of FG-4497 and G-CSF treatments (G+F). Flow-cytometry profiles on the left are overlays of CXCR4 protein expression with isotype matched control of CXCR4 antibody in shaded area, HSPCs from mice treated with G-CSF alone in blue, or with combination of G-CSF plus FG-4497 in red. The gray area represents fluorescence with APC-conjugated rat IgG2b Control. The middle and right plots represent cell surface CXCR4 mean fluorescence intensity and the proportion cells that are positive for CXCR4 respectively. Each dot represents an individual mouse. The bars are average for each group. (**b**) Intracellular staining for CXCR4 on Lin^−^Sca1^−^Kit^+^ BM HPCs (LKS-) and Lin^−^Sca1^+^Kit^+^ HSPCs (LKS+) from mice treated for 2 days with saline (S), or with FG-3347 for 3 days (F), or with G-CSF for 2 days (G), or the combination of FG-4497 and G-CSF treatments (G+F) as described in (**a**). (**c**) qRT-PCR for *Cxcr4* mRNA on Lin^−^Sca1^+^Kit^−^ cells sorted from the BM of mice treated with saline (S), FG-4497 (F), G-CSF (G) or combination of FG-4497 and G-CSF treatments (G+F). (**d**) Transwell migration of Lin^−^Sca1^+^Kit^+^ BM cells from mice treated for 2 days with G-CSF (black line and dots), or with FG-3347 for 3 days and G-CSF for the last 2 days, against 100 ng/ml CXCL12 in the bottom chamber. Each dot represents an individual mouse. The bars are average±s.d. Significance levels were calculated with Mann—Whitney test. (**e**) *Cxcl12* mRNA expression relative to β-actin in endosteal cells from mice treated with saline, FG-4497 for 3 days, G-CSF or 2 days or with FG-3347 for 3 days and G-CSF for the last 2 days. The chart shows median, 95% confidence intervals, maximum and minimum values from eight separate mice per treatment group. Significance levels were calculated with Dunn's test.

Mice were treated for 3 days of FG-4497 or saline, and with G-CSF for the last 2 days before BM harvest, a time point with highest blood mobilization difference between mice treated with G-CSF alone or with G-CSF plus FG-4497 (Figure 3b). Cell surface expression of CXCR4 was very low on BM Lin^−^Sca1^−^Kit^+^ HPCs or Lin^−^Sca1^+^Kit^+^ HSPCs from mice treated with G-CSF plus FG-4497 compared with mice treated with G-CSF alone in terms of both percentage of positive cells and fluorescence intensity for CXCR4 (Figure 4a). As an important pool of CXCR4 protein remains intracellular in stem and progenitor cells,^33^ we performed intracellular staining on fixed and permeabilized BM cells. Indeed a much higher proportion of Lin^−^Sca1^−^Kit^+^ HPCs or Lin^−^Sca1^+^Kit^+^ HSPCs stained positively for intracellular CXCR4. Treatment with FG-4497 significantly reduced CXCR4 intracellular staining with or without additional G-CSF treatment in terms of both percentage of positive cells and fluorescence intensity for CXCR4 (Figure 4b). qRT-PCR on Lin^−^ Sca1^+^ Kit^+^ HSPCs sorted from the BM of these treated mice showed that FG-4497 treatment or G-CSF treatment did not significantly alter *Cxcr4* mRNA expression by one-way ANOVA (Figure 4c). Taken together, this suggests that FG-4497 alters CXCR4 internalization or intracellular trafficking in HSPCs.

BM cells from these mice were also tested in a chemotaxis assay in the presence of 100 ng/ml CXCL12 in the bottom chamber. BM Lin^−^Kit^+^Sca1^+^ HSPC from mice treated with G-CSF plus FG-4497 migrated less toward CXCL12 compared with HSPC isolated from mice treated with G-CSF alone (Figure 4d), suggesting that FG-4497 treatment decreases CXCR4 signaling in HSPC from mobilized mice.

When repeated in mice not treated with G-CSF, 3-day FG-4497 treatment did not increase CXCR4 expression on HSPC isolated from mice in steady state. Likewise, the FG-4497 treatment did not alter transmigration toward CXCL12 (not shown). This may explain why FG-4497 alone does not mobilize HSPC *in vivo*.

We extracted RNA from endosteal cells from the femurs of mice treated with saline, FG-4497 alone, G-CSF alone or combination of G-CSF plus FG-4497. qRT-PCR revealed that *Cxcl12* mRNA was further decreased in the G-CSF plus FG-4497 combination group compared with G-CSF alone, suggesting that FG-4497 further reduce expression of CXCL12 by BM stromal cells in response to G-CSF (Figure 4e). Interestingly, in the absence of G-CSF, treatment with FG-4497 alone did not alter *Cxcl12* mRNA levels.

We also tested whether pharmacological stabilization of HIF-1α could alter adhesive interactions of HSPCs mediated by endothelial selectins, α4 and α5 integrins which are important to retain HSPC within the BM.^34, 35, 36^ FG-4497 did not reduce binding of recombinant mouse E-selectin or P-selectin-IgG1 fusion proteins to BM HSPCs, although G-CSF treatment significantly reduced P-selectin binding (Supplementary Figure S5). Cell surface expression of PSGL1, α4 and α5 integrins on BM HSPCs was not altered by *in vivo* treatments with FG-4497 (Supplementary Figure S5).

### Other PHD inhibitors enhance HSPC mobilization

**Figure 5:**
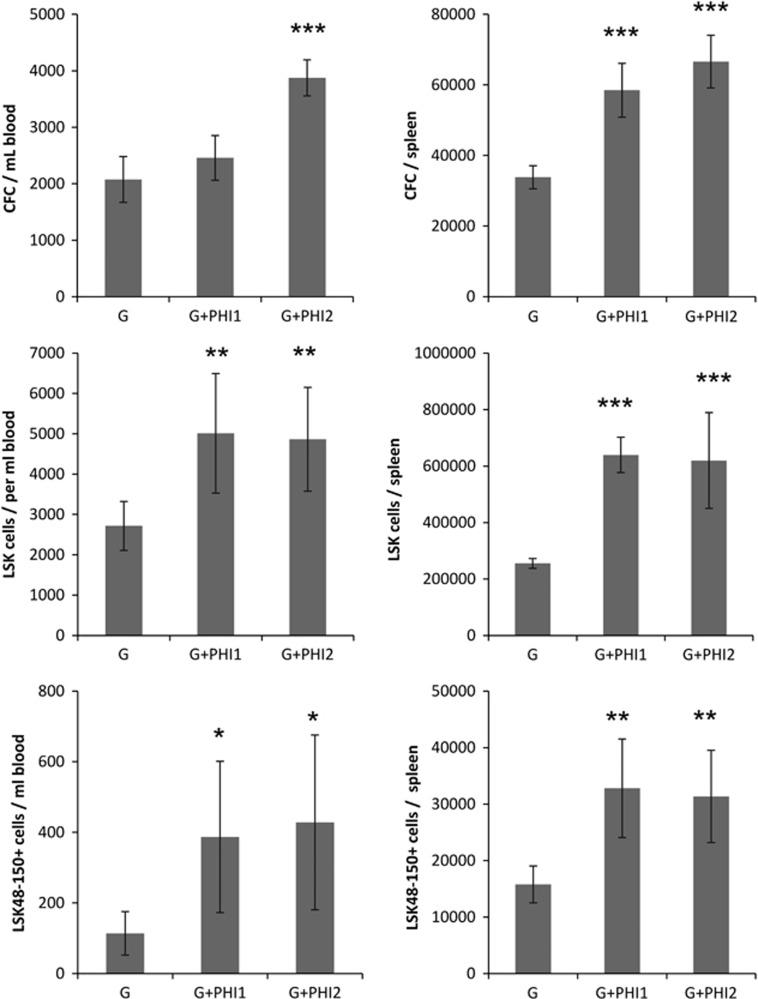
Two orally available PHD inhibitors enhance HSPC mobilization induced by G-CSF. C57BL/6 mice were injected subcutaneously twice daily with human G-CSF (125 μg/kg) for 4 consecutive days and gavaged once daily with vehicle (G), 20 mg/kg PHI-1(G+PHI1) or PHI-2 (G+PHI2) during the last 3 days of G-CSF injection. Mice were killed the next morning following that last G-CSF injection and numbers of CFCs, Lin^−^Sca1^+^ Kit^+^ (LSK) HSPCs and Lin^−^Sca1^+^Kit^+^CD48^-^CD150^+^ (LSK48- 150+) HSCs per ml of blood and per spleen measured. Data are mean±SD of six mice per group. *P-*values are relative to G-CSF treated group and were calculated with a Student's *t-*test. *P<0.05; **0.001<*P*<0.01; ***P<0.001.

To further document the potentiating effect of PHD inhibitors on HSPC mobilization, C57BL/6 mice were treated with two additional novel PHD inhibitors (PHI-1 and PHI-2) from chemically distinct structural classes (US patents number 7 696 223 and 7 928 120). Although representing different chemical series, FG-4497, PHI-1 and PHI-2 are all structural mimetics of the PHD substrate 2-oxoglutarate and inhibitors of the HIF PHD enzymes.^37^ Combinations of either PHI-1 or PHI-2 with G-CSF increased CFCs, Lin^−^Sca1^+^Kit^+^ HSPCs and Lin^−^Sca1^+^Kit^+^CD48^-^CD150^+^ HSCs mobilization into blood compared with G-CSF alone treated animals (Figure 5).

### PHD inhibition synergizes with the combination of G-CSF and Plerixafor

**Figure 6:**
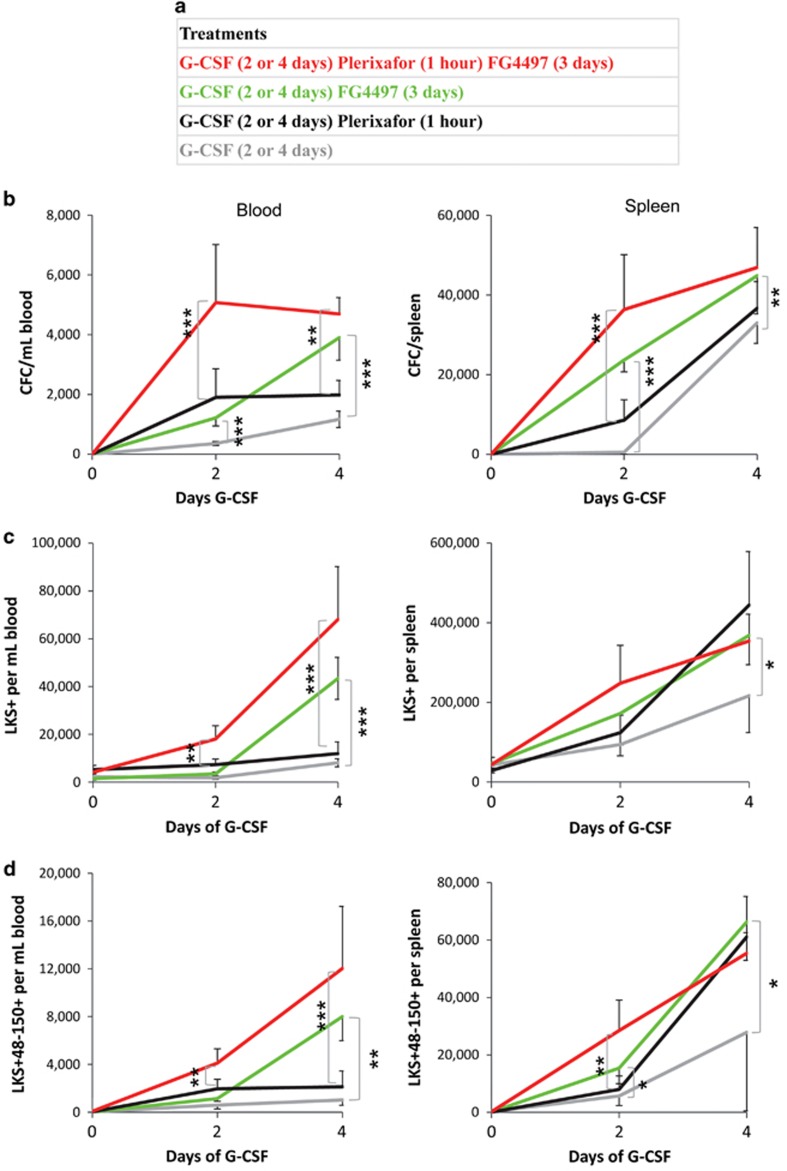
Synergistic effect of FG-4497 with G-CSF and Plerixafor. (**a**) Experimental scheme: Cohorts of mice were injected twice daily with G-CSF alone for 2 or 4 days (gray lines), or G-CSF for 2 or 4 days with one Plerixafor injection 1 h before tissue harvest (black lines). The effect of FG-4497 was tested by injecting mice daily with FG-4497 to mice injected twice daily with G-CSF for the last 2 or 4 days before tissue harvest (green lines). Alternatively FG-4497 was injected daily to mice injected twice daily with G-CSF for the last 2 or 4 days, and Plerixafor 1 h before tissue harvest (red lines). (**b**–**d**) Number of CFCs (**b**), Lin^−^Sca1^+^Kit^+^ HSPCs (**c**) and Lin^−^Sca1^+^Kit^+^CD48^-^CD150^+^ HSCs (**d**) per ml of blood or per spleen following treatments with combinations of G-CSF, Plerixafor and FG-4497 as in (**a**). Data are mean±s.d. of six mice per time point per treatment group. For statistical analyses, we compared (**a**) G-CSF alone group (gray line) to G-CSF with FG-4497 group (green line) and (**b**) G-CSF+Plerixafor group (black line) to G-CSF+Plerixafor+FG-4497 group (red line). Significant differences between treatment groups were calculated by one-way ANOVA with Bonferroni correction for multiple comparisons. Significant differences are indicated by brackets with the following symbols: *P<0.05; **0.001<*P*<0.01; ***P<0.001.

Since FG-4497 synergized with either G-CSF or Plerixafor, we tested whether FG-4497 could further enhance the mobilizing effect of the combination of G-CSF with Plerixafor. Mice were treated with G-CSF for 2 or 4 days, with or without Plerixafor 1 h before harvest or with or without FG-4497 for 3 days prior harvest (Figure 6a). The combination of G-CSF plus Plerixafor plus FG-4497 was much more potent at mobilizing CFCs, phenotypic HSPCs and HSCs to blood and spleen than the dual combination of G-CSF and Plerixafor (Figures 6b and d). At day 4 of mobilization, the numbers of CFCs, phenotypic HSPCs and HSCs mobilized into blood were 2.4-fold, 5.7-fold and 5.7-fold higher with G-CSF, Plerixafor and FG-4497 than with G-CSF plus Plerixafor without FG-4497. Of note, the dual combination G-CSF plus FG-4497 mobilized CFCs, HSPCs and HSCs into blood better than the dual combination of G-CSF plus Plerixafor although mobilization to the spleen was equivalent with these two regimens.

**Figure 7:**
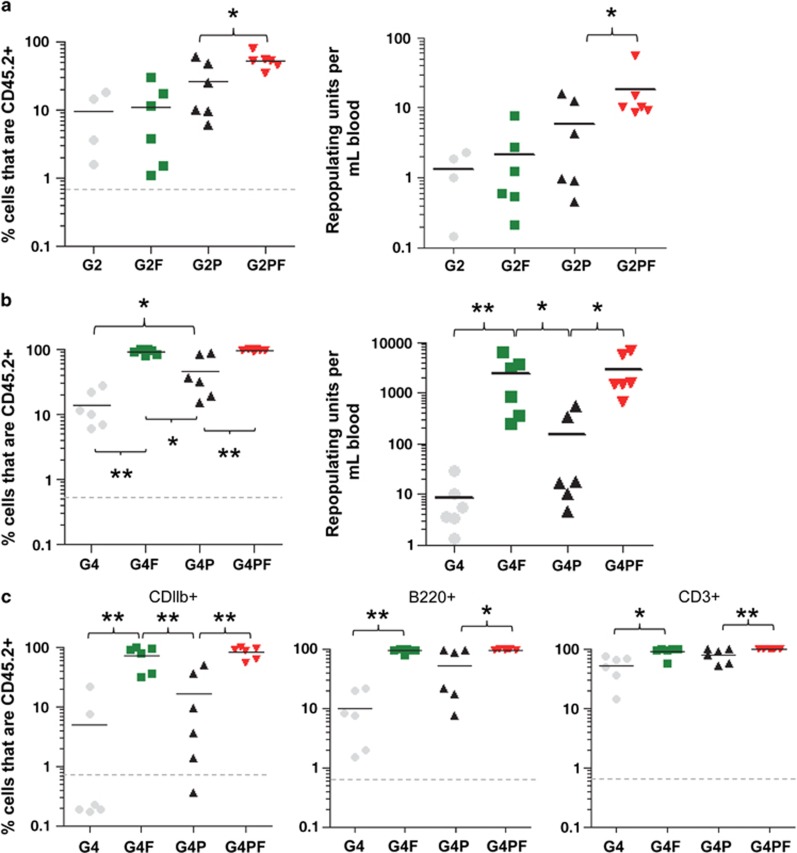
FG-4497 synergizes with G-CSF and Plerixafor to enhance mobilization of competitive repopulating HSCs. (**a**) CD45.2^+^ chimerism after transplantation of blood mobilized for 2 days with G-CSF, Plerixafor and FG-4497. CD45.2^+^ donors were mobilized with G-CSF alone for 2 days (G2), or G-CSF in combination with Plerixafor (G2P) or FG-4497 (G2F) or with both Plerixafor and FG-4497 (G2PF ) following the same schedule as in Figure 5a. 20 μl blood was transplanted in lethally irradiated recipients together with 200 000 competitive BM cells from CD45.1^+^ mice. Proportion of blood CD45.2^+^ leukocytes at 16 weeks post transplantation and number of repopulating units per ml of mobilized blood calculated from donor chimerism at 16 weeks post transplantation. (**b**) CD45.2^+^ chimerism after transplantation of blood mobilized for 4 days with G-CSF, Plerixafor and FG-4497. CD45.2^+^ donors were mobilized with G-CSF alone for 4 days (G4), or G-CSF in combination with Plerixafor (G4P) or FG-4497 (G4F) or with both Plerixafor and FG-4497 (G4PF ) following the same schedule as in Figure 5a. (**c**) CD45.2^+^ donor contribution was measured in blood 16 weeks post transplantation in total CD11b^+^ myeloid cells, B220^+^ B cells and CD3^+^ T cells by flow cytometry. Each dot is a result from one recipient mouse, bars are average, dotted lines represent 0.5% CD45.2^+^ threshold above which chimerism was considered as positive. *P<0.05; **0.001<*P*<0.01; ***P<0.001 using a non-parametric Mann–Whitney test.

This synergistic increase in HSC mobilization was further confirmed in long-term competitive repopulation assays following transplantation of 20 μl mobilized blood from CD45.2^+^ C57BL/6 mice in competition with 2 × 10^5^ BM cells from CD45.1^+^ B6.SJL congenic donors. CD45.2^+^ donor versus CD45.1^+^ competitor chimerism 16 weeks post transplantation confirmed that the tri-combination G-CSF plus Plerixafor plus FG-4497 mobilized competitive repopulating HSCs more than the dual combination G-CSF plus Plerixafor at both time points of G-CSF treatment *P*<0.01 (Figures 7a and b). Reconstitution with mobilized blood was significantly higher in all blood lineages (B, T and myeloid cells) (Figure 7c) with G-CSF plus Plerixafor plus FG-4497, mobilizing repopulating units 1.8-fold more at day 2, and 20-fold more at day 4 than the dual combination G-CSF plus Plerixafor (Figures 7a and b).

Long-term competitive transplantation assays also confirmed that G-CSF plus FG-4497 mobilized repopulating units 16-fold more than the combination of G-CSF plus Plerixafor at day 4 with higher multi-lineage chimerism.

## Discussion

Using conditional deletion of the *Hif1a* gene specifically in HSCs and immature HSPCs, we demonstrate that HIF-1α is required for HSPC mobilization in response to G-CSF or Plerixafor. More relevant to the clinic, we also demonstrate that pharmacological stabilization of HIF-1α protein with small synthetic PHD inhibitors synergizes with both G-CSF and Plerixafor to increase HSC mobilization into the blood. Indeed, additional stabilization of HIF-1α protein with the PHD inhibitor FG-4497 increased 20-fold the long-term repopulation potential of blood mobilized with G-CSF plus Plerixafor, currently the best available therapeutic combination to mobilize HSCs in autologous patients that previously failed to mobilize with G-CSF alone. This was not due to an off-target effect of FG-4497 as this drug did not enhance mobilization of HSPCs conditionally deleted for the *Hif1a* gene. Furthermore, other PHD inhibitors from different structural classes also enhanced HSPC mobilization similar to FG-4497. The synergistic effect of PHD inhibitors on HSC mobilization and engraftment upon transplantation is consistent with the recent finding that a short exposure *ex vivo* of HSPCs to dimethyl prostaglandin E2 stabilizes HIF-1α and subsequently doubles HSC homing to the BM and long-term engraftment.^18^ Therefore, *in vivo* treatment with PHD inhibitors may have the double advantage of (1) quantitatively increasing the number of HSCs mobilized into the blood and (2) increasing their subsequent homing and engraftment efficiency once transplanted.

Mechanistically, we show that *Hif1a* gene deletion inhibited HSPC mobilization in response to the CXCR4 inhibitor Plerixafor, and reduced their migration toward CXCL12 gradient without altering *Cxcr4* mRNA expression. However, due to the extremely low levels of CXCR4 protein at the surface of mouse HSPCs in the BM, we could not detect significant changes in CXCR4 protein at the surface of HSPCs following *Hif1a* gene deletion. Because of this we could not detect changes in cell surface CXCR4 protein on HPSCs from mice treated with FG-4497. However, treatment of wild-type mice with FG-4497 alone or in combination with G-CSF, which further stabilizes HIF-1α protein, significantly reduced intracellular CXCR4 protein in HSPCs compared with mice treated with saline or with G-CSF alone. However, FG-4497 treatment did not alter *Cxcr4* mRNA expression in HSPCs. This is consistent with previous findings showing that deletion of *Hif1a* gene in all hematopoietic cells and in stromal cells in *Mx1*-Cre *Hif1a*^*fl/fl*^ mice did not alter *Cxcr4* mRNA or CXCR4 protein surface expression on HSPCs.^17^ In addition, we found that *in vitro* chemotaxis of BM HSPCs isolated from mice treated with G-CSF plus FG-4497 was also decreased compared with HSPCs from mice treated with G-CSF alone. This is in contrast with a recent report that *ex vivo* treatment of BM HSPCs with dimethyl prostaglandin E2, which also increases HIF-1α protein, enhanced HSPC transmigration toward CXCL12.^18^ A possible reason for this discrepancy is that dimethyl prostaglandin E2 was used in *ex vivo* once HSCs removed from their physiological niches while in our experiments, FG-4497 was administered *in vivo*. Also, dimethyl prostaglandin E2 is likely to activate via its four receptors EP1-4 many other pathways in addition to HIF-1α.^38^ Finally, we found that the combination of FG-4497 plus G-CSF further exacerbated the reduction of *Cxcl12* mRNA expression in the BM stroma in response to G-CSF. Therefore, the effect of FG-4497 is likely mediated in part by perturbation of CXCR4 signaling (HSPC autonomous effect) and CXCL12 expression (extrinsic effect) in G-CSF-treated mice, which may ultimately lead to enhanced mobilization. In summary, our data suggest that in G-CSF-treated mice, HIF-1α stabilization with FG-4497 qualitatively alters or quantitatively decreases CXCR4 chemotactic signaling. However, as HIF-1α activates the transcription of hundreds of genes,^14, 39^ it is likely that HIF-1α enhances HSPC mobilization via several parallel mechanisms. Therefore, the effect of HIF-1α stabilization on HSPC mobilization is likely to be complex resulting from both HSC autonomous mechanisms and stroma mediated-mechanisms. Ongoing studies in our laboratory to profile the transcriptome of HSPCs from mice treated with FG-4497 will provide insights about its additional mechanisms of action on HSPC mobilization.

If corroborated in humans, then our current study suggests that PHD inhibitors such as FG-4497 could advantageously replace Plerixafor or be added to G-CSF and Plerixafor to form an even more potent mobilizing cocktail that could further reduce the numbers of patients who fail to mobilize sufficiently to proceed with autologous HSC transplantation. Indeed, PHD inhibitors are safe in humans and currently tested in several clinical trials to treat anemia associated with chronic renal diseases.^37^ Therefore, PHD inhibitors may have the potential to (1) reduce the high cost of remobilization^40, 41^ and make it more accessible to patients requiring autologous HSC transplant and (2) be used upfront with G-CSF to increase the number of HSCs harvested in both the allogeneic and autologous setting as transplantation of higher doses of HSCs (>5 × 10^6^ CD34^+^ cells/kg) further reduce the period of cytopenia, risks of potentially fatal febrile neutropenia, and transfusion costs associated with prolonged anemia and thrombocytopenia.

In conclusion, our work demonstrates that HIF-1α is necessary to HSPC mobilization in mice and opens the perspective of using PHD inhibitors in combination with G-CSF to boost HSC mobilization for transplantation in humans.

## Notes

GW is an employee of and owns equity in FibroGen Inc., which owns the commercial rights to FG-4497, PHI-1 and PH-2. FibroGen provided funding for experiments with PHI-1 and PHI-2. JPL and IGW are the inventors of a US patent describing these results.
