# Supplementary material for: HIF-1α is required for hematopoietic stem cell mobilization and 4-prolyl hydroxylase inhibitors enhance mobilization by stabilizing HIF-1α
Source: Leukemia. 2015 Feb 3;29(6):1366–78. doi: 10.1038/leu.2015.8 (PMC4498452; doi:10.1038/leu.2015.8)
Supplement: Supplementary Figures [file leu20158x1.pdf]

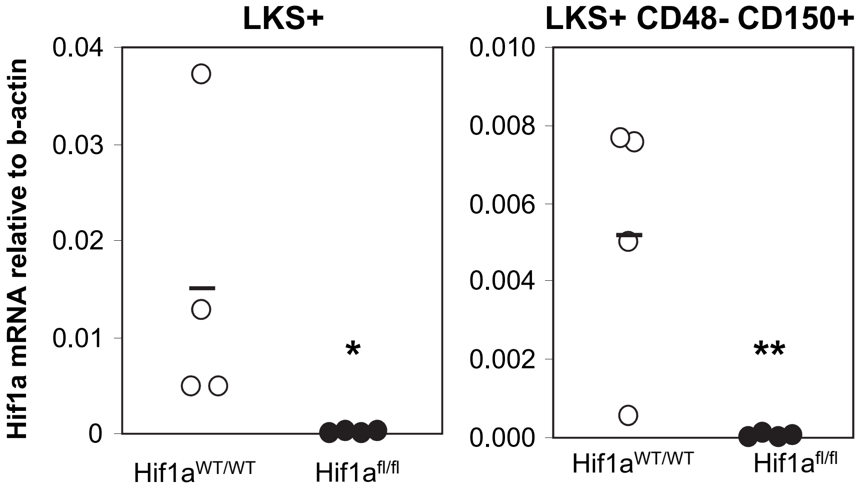

Figure S1. Intact Hif1a mRNA is depleted in SclCreER R26R$^{YFP/YFP}$ Hif1a$^{fl/fl}$ mice. SclCreER R26R$^{YFP/YFP}$ *Hif1a*$^{fl/fl}$ and SclCreER R26R$^{YFP/YFP}$ *Hif1a*$^{WT/WT}$ mice were gavaged daily with tamoxifen for 3 days to induce CreER. Lin$^-$Sca1$^+$Kit$^+$ YFP$^+$ HSPCs (left panel) and Lin-Sca1+Kit+CD48-CD150+ YFP+ HSCs (right panel) were sorted from SclCreER R26R$^{YFP/YFP}$ *Hif1a*$^{fl/fl}$ or SclCreER R26R$^{YFP/YFP}$ *Hif1a*$^{WT/WT}$ mice, RNA extracted and qRT-PCR for exon 2-3 of Hif1a mRNA performed. Each dot represents cells sorted from a separate mouse. Bars are the average for each group. * symbols show significant statistical differences ($p<0.05$) between the two genotypes.

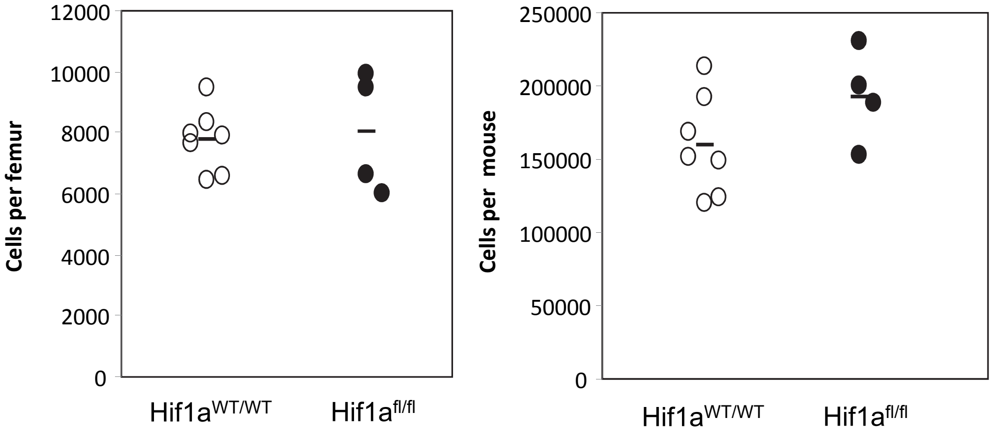

Figure S2. The number of phenotypic HSCs in femoral BM and mice is not altered by *HIF1a* gene deletion. SclCreER R26R$^{YFP/YFP}$ *Hif1a*$^{fl/fl}$ and SclCreER R26R$^{YFP/YFP}$ *Hif1a*$^{WT/WT}$ mice were gavaged daily with tamoxifen for 3 days to induce CreER, and then injected twice daily with G-CSF for the last 3 days prior tissue sampling. Left panel id the number of phenotypic Lin^{-} Sca1^{+} Kit^{+} CD48^{-} CD150^{+} HSCs per femoral BM. Right panel is the total number of phenotypic HSCs in total BM, blood and spleen after summation of content in BM, blood and spleen. Each dot represents an individual mouse and bars are averages for each genotype.

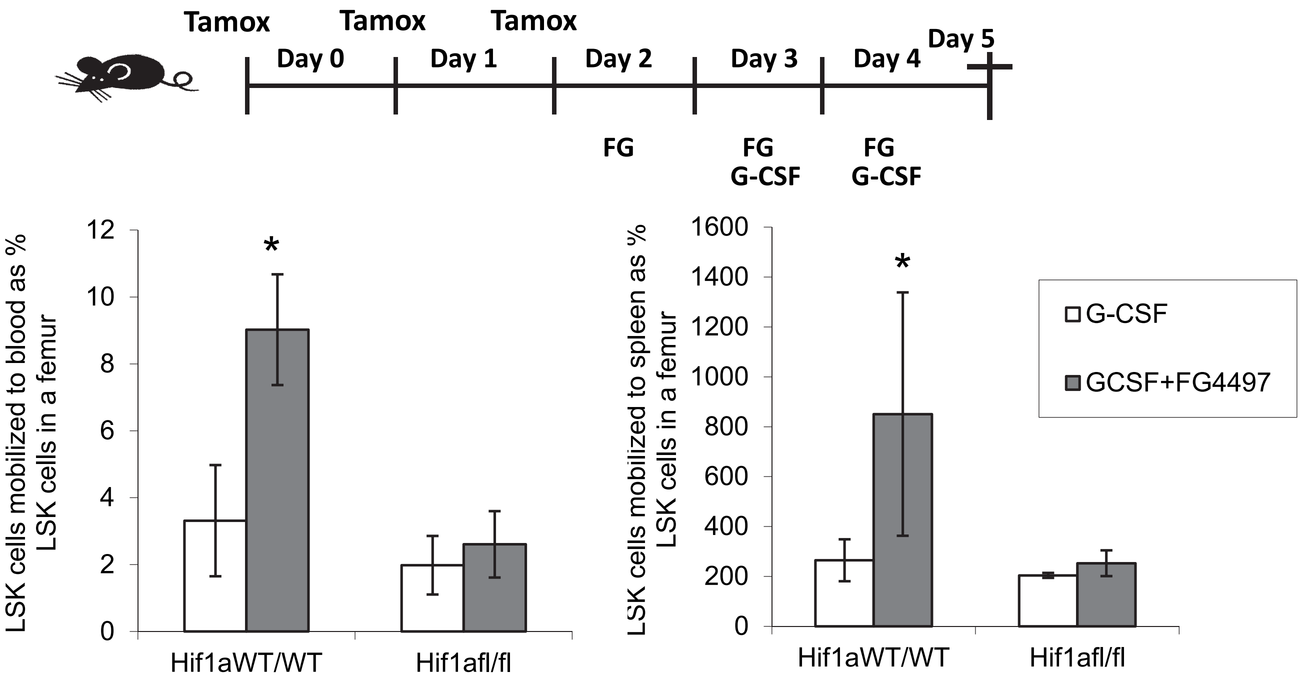

Figure S3. FG-4497 effect on HSC mobilization is mediated by HIF-1α. FG-4497 does not enhance mobilization of *Hif1a*-deficient HSPCs. SclCreER R26R$^{YFP/YFP}$ *Hif1a*$^{fl/fl}$ and SclCreER R26R$^{YFP/YFP}$ *Hif1a*$^{WT/WT}$ mice were gavaged daily with tamoxifen for 3 days to induce CreER, and then injected daily with FG-4497 for the last 3 days, and twice daily with G-CSF for the last 2 days prior tissue sampling. Proportion of Lin^{-}Sca1^{+}Kit^{+} HSPCs from SclCreER R26R$^{YFP/YFP}$ *Hif1a*$^{fl/fl}$ or SclCreER R26R$^{YFP/YFP}$ *Hif1a*$^{WT/WT}$ mice mobilized into the blood or spleen after induction with tamoxifen and subsequent treatment with G-CSF alone or G-CSF + FG-4497. Data are expressed as percentage of Lin^{-}Sca1^{+}Kit^{+} HSPCs per ml of blood or per spleen relative to the number of Lin^{-}Sca1^{+}Kit^{+} HSPCs in whole BM of a femur. Data are mean ± SD of 3 to 4 mice per group. * symbols show significant statistical differences ($p<0.05$) between G-CSF group and G-CSF+FG-4497 treatment groups with a specific genotype.

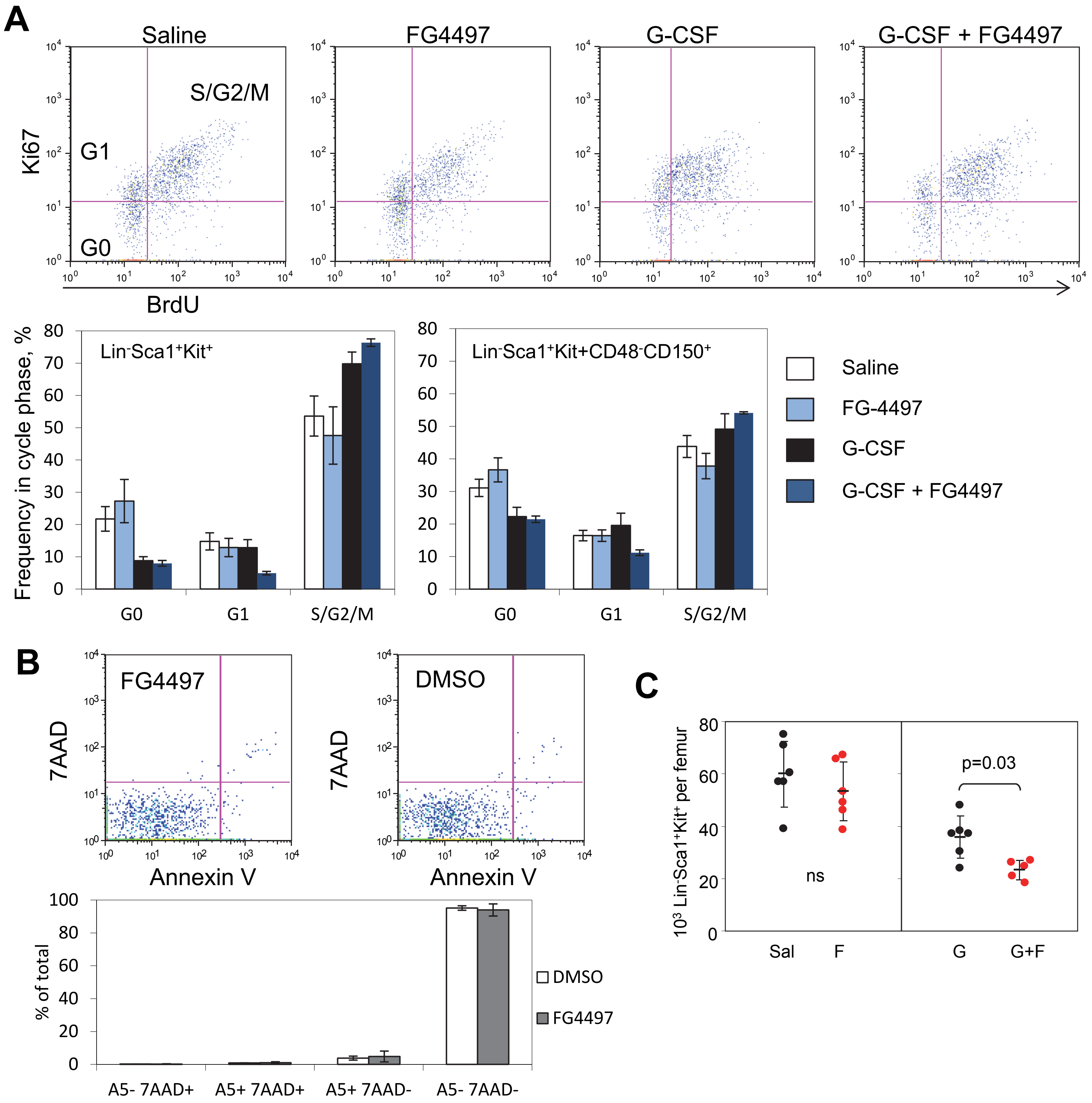

Figure S4. Mobilizing effect of FG-4497 on mobilization does not involve changes in HSC cycling or survival. (A) A 3 day treatment with FG-4497 does not alter HSPC cycling in the BM. Mice had BrdU in drinking water during the 3 days of the experiment and were treated with either saline, FG-4497 for 3 days, G-CSF for last 2 days or the combination of G-CSF and FG-4497. Dot-plots show typical Ki67 and BrdU staining in BM $Lin^-Sca1^+Kit^+$ HSPCs from each group. Histograms show proportions of $Lin^-Sca1^+Kit^+$ HSPCs and $Lin^-Sca1^+Kit^+CD48^-CD150^+$ HSCs in different phases of cell cycles in each treatment group. Data are mean ± SD of 4 mice per group. (B) Ex vivo treatment with FG-4497 does not alter HSPC survival. Whole BM cells were cultured overnight with 10ng/ml kit ligand and 40μM FG-4497 or 0.2% DMSO (vehicle). On the next day 7AAD versus Annexin V (A5) binding was measured by flow cytometry on $Lin^-Sca1^+Kit^+CD48^-$ HSCs. Data are mean ± SD of 3 separate mice per group. (C) FG-4497 treatment does not increase $Lin^-Sca1^+Kit^+$ HSPC number in the BM. Mice were treated with either saline, FG-4497 for 3 days, G-CSF for 2 days, or the combination of G-CSF and FG-4497. The number of $Lin^-Sca1^+Kit^+$ HSPC was quantified in the femoral BM by flow cytometry. Each dot shows a separate mouse, bars are mean ± SD. Significance was calculated with Mann-Whitney test.

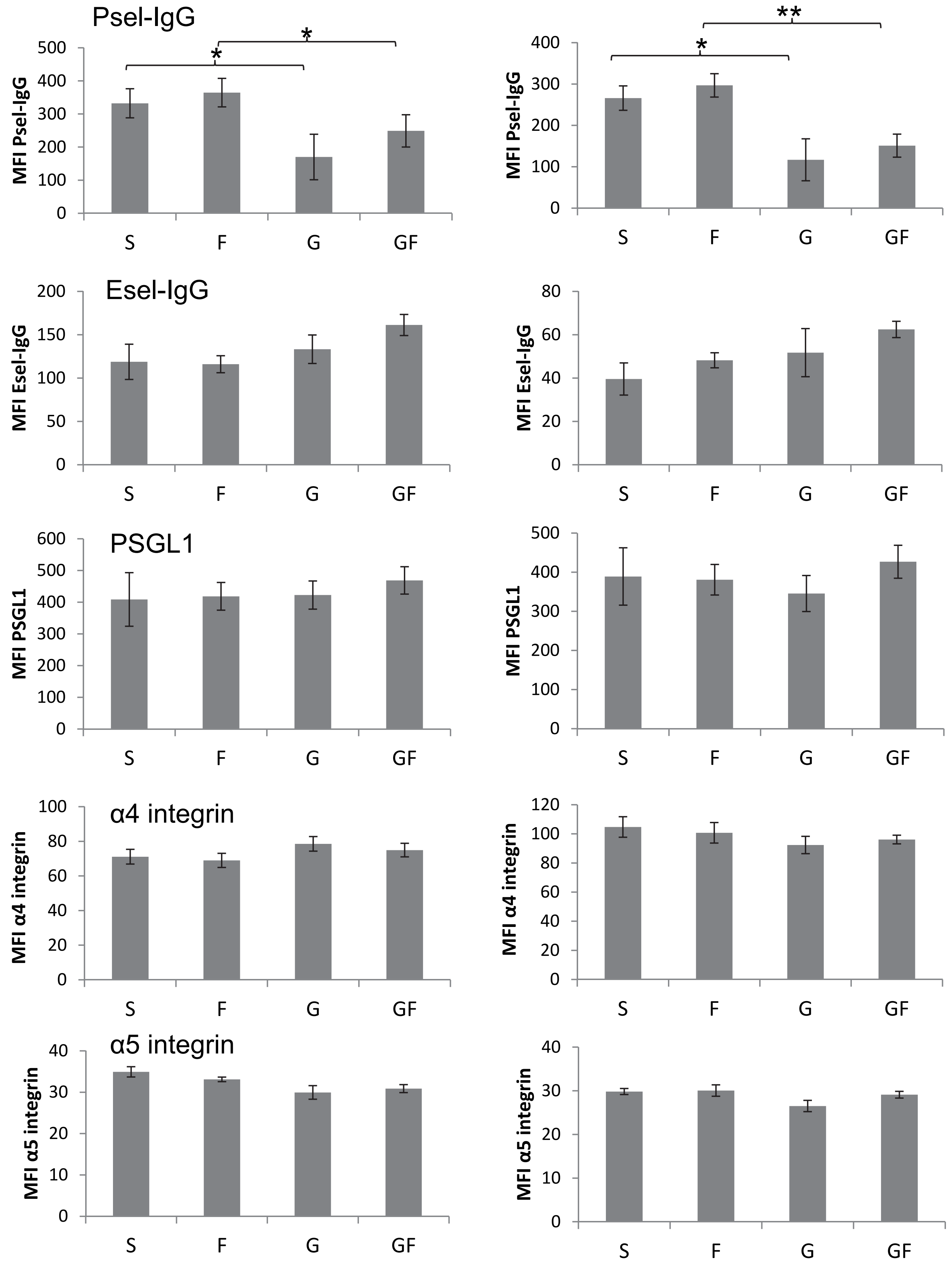

Figure S5. In vivo treatment with FG-4497 does not alter binding of selectins or expression of PSGL-1, α4- and α5 integrins on bone marrow HSPCs. Bone marrow cells from mice treated for 4 days with saline (S), FG-4497 (F), G-CSF (G) or G-CSF + FG-4497 (GF) and stained with either recombinant mouse E-selectin-human IgG1 Fc (Esel IgG), or E-selectin-human IgG1 Fc (Psel IgG) fusion proteins, or rat anti-mouse PSGL1, anti-mouse α4-integrin or anti-mouse α5 integrin. Fluorescence was measured on $Lin^-Sca1^+Kit^+$ HSPCs (left column of histograms) and $Lin^-Sca1^+Kit^+CD48^-CD150^+$ HSCs (right column of histograms). Data represent the specific mean fluorescence intensity minus fluorescence from controls. For selectin fusion proteins, negative controls were identical bone marrow cells incubated with same fusion protein but in the presence of 5mM EDTA. For monoclonal antibodies, negative controls were incubated with isotype matched non immune rat IgG monoclonal antibodies. Data are mean ± SD of 4 mice per treatment group.
