# Supplementary material for: HIF-1α is required for hematopoietic stem cell mobilization and 4-prolyl hydroxylase inhibitors enhance mobilization by stabilizing HIF-1α
Source: Leukemia. 2015 Feb 3;29(6):1366–78. doi: 10.1038/leu.2015.8 (PMC4498452; doi:10.1038/leu.2015.8)
Supplement: Supplementary Table S1 [file leu20158x2.pdf]

Table S1: Antibodies used for flow cytometry

Antibodies	Clones	Suppliers	Dilutions
CD3ε-biotin	145-2C11	BioLegend	1/300
B220-biotin	RA3-6B2	BioLegend	1/300
CD11b-biotin	M1/70	BioLegend	1/300
Gr1-biotin	RB6-8C5	BioLegend	1/300
Ter119-biotin	Ter119	BioLegend	1/300
CD41-biotin	MWreg30	BD Pharmingen	1/300
Sca1-PECY7	D7	BioLegend	1/300
Sca1-PE	D7	BioLegend	1/300
Kit(CD117)-APC	2B8	BioLegend	1/300
CD48-Pacific Blue	HM48-1	BioLegend	1/200
CD150-PE	TC15-12F 12.2	BioLegend	1/300
Kit(CD117)-APCy7	2B8	BD Pharmingen	1/200
CD34-FITC	RAM34	BD Pharmingen	1/75
CD48-PerCPCy5.5	HM48-1	BioLegend	1/150
α4 integrin-FITC	R1-2	BioLegend	1/200
α5 integrin-A647	5H10-27(MFR5)	BioLegend	1/200
PSGL1-BV421	2PH1	BD Pharmingen	1/300
CXCR4-APC	L276F12	BioLegend	1/200
CXCR7-APC	8F11-M16	BioLegend	1/20
Ki67-A700	B56	BD Pharmingen	1/10
BrdU-FITC		BD Pharmingen	As per kit instructions
SAV-BV605		BioLegend	1/300
