# Supplementary material for: HIF-1α is required for hematopoietic stem cell mobilization and 4-prolyl hydroxylase inhibitors enhance mobilization by stabilizing HIF-1α
Source: Leukemia. 2015 Feb 3;29(6):1366–78. doi: 10.1038/leu.2015.8 (PMC4498452; doi:10.1038/leu.2015.8)
Supplement: Supplementary Methods [file leu20158x3.pdf]

SUPPLEMENTARY METHODS

Mouse genotyping

Scl-CreER R26RYFP/YFP Hif1aflox/flox mice with HSC specific tamoxifen-inducible deletion of the Hif1a gene and induction of the YPF reporter, and control Scl-CreER R26RYFP/YFP mice with two Hif1a wild-type alleles were produced by intercrossing the three parental strains. Offspring were genotyped from ear clips by PCR using the following primers: R26RYFP allele (5'-GCGAAGAGTTTGTCCTCAACC-3' and 5'-AAAGTCGCTCTGAGTTGTTAT-3'), $R26R^{WT}$ allele (5'-AGTAAGGATCTCAAGCAGGAG-3') $R26R^{YFP}$ allele 250bp, WT allele 500bp; Cre transgene (5'-GAGTGATGAGGTTCGCAAGA-3' and 5'-CTACACCAGAGACGGAAATC-3', 615bp), Hif1a alleles (5'-CAAGCATTCTTAAATGTGGAGC-3' and 5'-GGGCAGTACTGGAAAGATGG-3'; WT allele 256 bp; floxed allele 300 bp).

Flow cytometry staining

Following flushing with PBS containing 2% FCS, enriched central BM cells were pelleted and resuspended in CD16/CD32 hybridoma 2.4G2 supernatant to block IgG Fc receptors. HSPCs were stained with the biotinylated lineage antibody cocktail (CD3, CD5, B220, CD11b, Gr-1, Ter119) and biotinylated CD41 together with streptavidin (SAV)-Brilliant Violet 605 (BV605), anti-Kit-allophycocyanin (APC), CD48-PacBlue and CD150-phycoerythrin (PE) anti-Sca-1-PE-cyanin 7 (PECY7) as previously described[1]. To measure expression of cell adhesion receptors cells were stained with the biotinylated lineage antibody cocktail (CD3, CD5, B220, CD11b, Gr-1, Ter119) and biotinylated CD41 together with streptavidin (SAV)-BV605, anti-Sca-1-PECY7, anti-Kit-APC-Cy7, CD48-PercpCy5.5, CD150-PE, anti-α4 integrin-fluoroscein isothiocyanate (FITC), anti-α5 integrin-Alexa 647 (A647) and anti-PSGL1-PacBlue. For CXCR4 and CXCR7 staining, HSCs were stained with the biotinylated lineage antibody cocktail (CD3, CD5, B220, CD11b, Gr-1, Ter119) and biotinylated CD41 together with streptavidin (SAV)-BV605, anti-Sca-1-PE, anti-KIT-APC-Cy7, CD48-PacBlue, CD150-PE and anti-CXCR4-APC. Rat IgG2b-APC antibody was used as isotype control for CXCR4 staining, and a mouse IgG2b-APC for CXCR7 staining. For intracellular CXCR4 staining, cells were labeled for extracellular antigens as described above, then washed fixed and permeabilized as previously described[2] before staining with anti-CXCR4-APC or rat IgG2b-APC. Antibody clones are detailed in Table S1.

For selectin binding, HSCs were stained as above for and then incubated with 5µg/ml recombinant mouse Eselectin-IgG1 Fc chimera or 1µg/ml Pselectin-IgG1 Fc chimera (R&D Systems) pre-complexed with 5 µg/ml A647-conjugated donkey $F(ab)_2$ fragment anti-human IgG (Jackson Immunoresearch) in Xvivo-10 medium for 2 hours as previously described[3]. 10mM EDTA was added to block binding in negative controls.

For BrdU incorporation and Ki67 staining, mice were given 0.5 mg/mL BrdU in their drinking water for the last 3 days prior tissue sampling in addition with their treatments with FG4497 and or G-CSF. BM cells were cell surface stained, then fixed and permeabilized before staining with FITC-conjugated anti-BrdU and AlexaFluor700-conjugated anti-Ki67 monoclonal antibodies as previously described[2,4].

Data were acquired on a CyAn 9C (Beckman Coulter) or a LSRII (BD Biosciences) flow cytometer and analyzed following compensation with single color controls using FlowJo software (Tree Star, Ashland, OR).

qRT-PCR

Hif1a gene deletion was confirmed by qRT-PCR on RNA samples extracted from Lin^- $Sca1^+Kit^+$ YFP^+ HSPCs from *Scl*-CreER $R26R^{YFP/YFP}$ *Hif1a*$^{flox/flox}$ and *Scl*-CreER $R26R^{YFP/YFP}$ *Hif1a*$^{WT/WT}$ mice. After a 5 day gavage with tamoxifen as described above, BM was sampled from hips, femurs and tibias, crushed in PBS containing 0.2% new-born calf serum. BM cell suspension was enriched in Kit^+ cells by magnetic activated cell sorting using mouse CD117 magenitic beads, and the magnetic fraction was stained with biotinylated anti-linage cocktail (CD3ε, B220, CD11b, Gr1, Ter119), anti-Sca1-PECY7 and anti-Kit-APC as described[2]. 10,000 $Lin^-Sca1^+Kit^+$ YFP^+ HSPCs were sorted from each mouse directly into 1ml of TRIzol (Life Technologies) to extract RNA. RNA was extracted following manufacturer's instructions, reverse transcribed using the iScript cDNA synthesis kit (Bio-Rad) and amplified by qRT-PCR using SYBR® Green PCR Master Mix (Life Technologies) with forward primer in *Hif1a* exon 2 5'-TGCTCATCAGTTGCCACTTC-3' and reverse primer in exon 3, 5'-CCATCTGTGCCTTCATCTCA-3' (10s at 95°C, 45s at 60°C per cycle) on a on ABI 7900 real-time thermocycler. For *Cxcr4* mRNA expression, forward primer 5'-ATGGAACCGATCAGTGTGAGTA-3' and reverse primer 5'-GTAGATGGTGGGCAGGAAGA-3' were used. Results were normalized on mouse β-actin mRNA using 5'-AGC ACT GTG TTG GCA TAG AGG TC-3' forward primer and 5'- CTT CTT GGG TAT GGA ATC CTG TG-3' reverse primer. For detection of Cxcr7 mRNA we used commercial primers and fluorescent probe TaqMan® Gene Expression Assays sets

(Mm00432610_m1 for mouse Cxcr7 and Mm01205647_g1 for β-actin) together with TaqMan® Universal PCR Master Mix no AmpErase® from Life Technologies.

RNA was extracted from endosteal cells from the femurs of mobilized mice and *Cxcl12* mRNA was then quantified by qRT-PCR relative to β-actin as described[5].

REFERENCES

1 Barbier V, Winkler IG, Levesque JP. Mobilization of hematopoietic stem cells by depleting bone marrow macrophages. *Methods Mol Biol* 2012; **904:** 117-138.

2 Barbier V, Nowlan B, Levesque JP, Winkler IG. Flow cytometry analysis of cell cycling and proliferation in mouse hematopoietic stem and progenitor cells. *Methods Mol Biol* 2012; **844:** 31-43.

3 Brooke G, Tong H, Levesque JP, Atkinson K. Molecular trafficking mechanisms of multipotent mesenchymal stem cells derived from human bone marrow and placenta. *Stem Cells Dev* 2008; **17:** 929-940.

4 Winkler IG, Barbier V, Nowlan B, Jacobsen RN, Forristal CE, Patton JT *et al.* Vascular niche E-selectin regulates hematopoietic stem cell dormancy, self renewal and chemoresistance. *Nat Med* 2012; **18:** 1651-1657.

5 Winkler IG, Sims NA, Pettit AR, Barbier V, Nowlan B, Helwani F *et al.* Bone marrow macrophages maintain hematopoietic stem cell (HSC) niches and their depletion mobilizes HSCs. *Blood* 2010; **116:** 4815-4828.
